# The complete chloroplast genome sequence of *Goodyera foliosa*（Orchidaceae）

**DOI:** 10.1080/23802359.2019.1674728

**Published:** 2019-10-11

**Authors:** Jie Zhou, Tai-Xiang Xie, Shan-Hu Ma, Ming-Kun Chen, Qing-Dong Zheng, Ye Ai

**Affiliations:** Key Laboratory of National Forestry and Grassland Administration for Orchid Conservation and Utilization at College of Landscape Architecture, Fujian Agriculture and Forestry University, Fuzhou, China

**Keywords:** *Goodyera foliosa*, chloroplast genome, orchid, phylogenetic analysis

## Abstract

*Goodyera foliosa* is a terrestrial orchid in Asia and has been listed as an endangered species in the Red List. In this study, we assembled the complete chloroplast genome of *G. foliosa* using Illumina sequencing data. Its full-length of 154,008 bp including a pair of invert repeats (IR) regions of 25,045 bp, large single-copy (LSC) region of 83,248 bp, and small single-copy (SSC) region of 20,670 bp. The chloroplast genome contains 127 genes, including 80 protein-coding genes, 39 tRNA genes, and 8 rRNA genes. In addition, the phylogenetic analysis base on 12 chloroplast genomes of Orchidaceae indicates that *G. schlechtendaliana* is closely related to *G. foliosa*. Our study would be helpful for the formulation of conservation strategies and further research of *G. foliosa.*

The genus *Goodyera* (Orchidaceae) comprises 40 species and is widely distributed in Madagascar, Asia, South Africa, northeast Australia, Europe, Madagascar, North America and the Southwest Pacific islands (Chen et al. 2009; Hu et al. 2016). Most species of the genus *Goodyera* are terrestrial (rarely epiphytic) orchid, which grows on mossy rocks or the moist banks of mountains (Pridgeon et al. 2003). *Goodyera foliosa* is a terrestrial orchid, growing under forests at an altitude of 300 and 1500 m (Zha et al. 2016, He et al. 2018). However, due to human activities and climate change, the habitat of *G. foliosa* has been lost or fragmented and wild populations sharply decreased in recent years. As a result, *G. foliosa* has been listed as an endangered species in the Red List (IUCN 2018). In this study, we assembled the complete chloroplast genome of *G. foliosa*, which would be helpful for the formulation of conservation strategies and further research.

The plant material of *G. foliosa* was collected from Qinglong waterfall scenic area, Yongtai, Fujian province, China (25°46′23.34″N, 118°57′50.55″E). The voucher specimen is kept at Herbarium of Fujian Agriculture and Forestry University (specimen code FAFU08012).

The total genomic DNA was extracted from fresh leaves using the modified CTAB method (Doyle and Doyle 1987) and sequenced based on the Illumina pair-end technology. Approximately 5 Gb of sequences data were extracted from the total sequencing output and input into Organelle PBA (Soorni et al. 2017) to assemble the chloroplast genome. Annotation of the chloroplast genome was performed using the Dual Organellar GenoMe Annotator (DOGMA) online tool (Wyman et al. 2004) and Geneious ver. 2019.1.1 (Li et al. 2019), then manually verified and corrected by comparison with *G. procera* (GenBank accession NC.029363). Finally, we obtained a complete chloroplast genome of *G. foliosa* and submitted to GenBank with accession number (MN.443774).

The total chloroplast genome sequence of *G. foliosa* is 154,008 bp in length and has a GC content of 37.3%. It contains a pair of inverted repeats (IR) regions of 25,045 bp, a large single-copy (LSC) region of 83,248 bp, and a small single-copy (SSC) region of 20,670 bp. Besides, the chloroplast genome contains 127 genes, including 80 protein-coding genes, 39 tRNA genes, and 8 rRNA genes.

To reveal the phylogenetic position of *G. foliosa* with other members of Orchidaceae, a phylogenetic analysis was performed based on 12 complete chloroplast genomes of Orchidaceae (*G. schlechtendaliana*, *G. foliosa*, *G. fumata*, *G. procera*, *Ludisia discolour*, *Anoectochilus emeiensis*, *Goodyera velutina*, *Habenaria radiata*, *Habenaria pantlingiana*, *Platanthera japonica*, *Cypripedium formosanum*, *Paphiopedilum niveum*). All the sequences were downloaded from NCBI GenBank. The sequences were aligned by MAFFT v7.307 (Katoh and Standley 2013), and the phylogenetic tree was constructed by RAxML (Stamatakis 2014). The results showed that *G. foliosa* was most closely related to *G. schlechtendaliana* with 100% bootstrap support (Figure 1).

**Figure 1.**
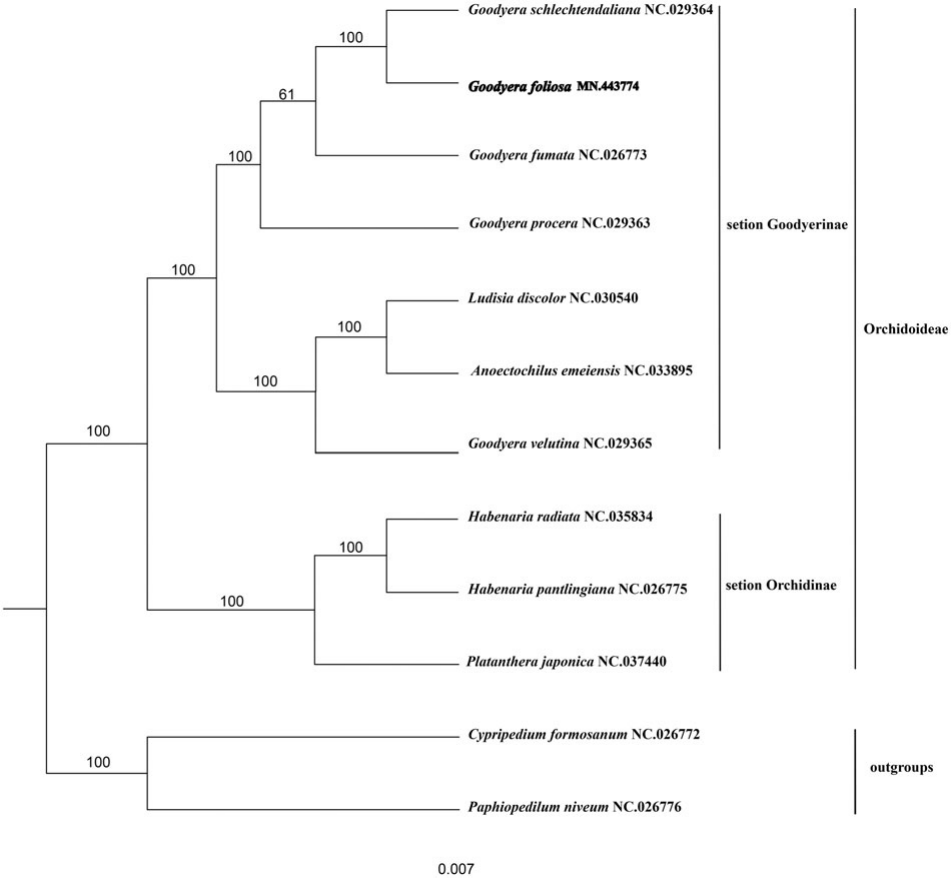
A phylogenetic tree was constructed based on 12 complete chloroplast genome sequences of Orchidaceae. All the sequences were downloaded from NCBI GenBank.
